# The annual rate of coronary artery calcification with combination therapy with a PCSK9 inhibitor and a statin is lower than that with statin monotherapy

**DOI:** 10.1038/s41514-018-0026-2

**Published:** 2018-06-22

**Authors:** Yuichi Ikegami, Ikuo Inoue, Kaiji Inoue, Yuichi Shinoda, Shinichiro Iida, Seiichi Goto, Takanari Nakano, Akira Shimada, Mitsuhiko Noda

**Affiliations:** 1 Department of Endocrinology and Diabetology, Saitama Medical University, 38 Moroyama, Iruma-gun, Saitama 350-0495 Japan; 2 Department of Radiology, Saitama Medical University, 38 Moroyama, Iruma-gun, Saitama 350-0495 Japan; 3 Department of Rehabilitation, Ibaraki Rehabilitation Hospital, 360 moriya-shi, Ibaraki, 302-0112 Japan; 4 Health Management Center, Saitama Medical University, 38 Moroyama, Iruma-gun, Saitama 350-0495 Japan; 5 Department of Biochemistry, Saitama Medical University, 38 Moroyama, Iruma-gun, Saitama 350-0495 Japan

**Keywords:** Diseases, Medical research, Geriatrics

## Abstract

Statins and/or PCSK9 inhibitors cause the regression of coronary atheroma and reduce clinical events. However, it currently remains unclear whether these drugs modulate coronary atheroma calcification in vivo. Coronary artery calcium (CAC) scores (Agatston Units, AUs) were estimated in 120 patients receiving coronary computed tomographic angiography (CCTA) (63% males; median age 56 years). The CAC scores were compared among the three groups: (1) neither statin nor PCSK9 inhibitor therapy, (2) statin monotherapy, and (3) statin and PCSK9 inhibitor combination therapy in an unpaired cross-sectional study. Additionally, CCTA was performed twice at an interval in 15 patients undergoing statin monotherapy to compare the previous (baseline) and subsequent (follow-up) CAC scores in a paired longitudinal study. In addition, a PCSK9 inhibitor was administered to 16 patients undergoing statin therapy. Before and after that, CCTA was performed twice to compare the previous and subsequent CAC scores in a paired longitudinal study. The unpaired cross-sectional study and paired longitudinal study consist of completely different patients. Among 120 patients, 40 (33%) had a CAC score >100 AUs. The median CAC score increased in the following order: statin group, statin and PCSK9 group, and no-statin-no-PCSK9 group. Annual CAC score progression was 29.7% by statin monotherapy and 14.3% following the addition of the PCSK9 inhibitor to statin therapy. The annual rate of CAC with the combination therapy with a PCSK9 inhibitor and a statin is lower than that with statin monotherapy. CAC may be prevented with PCSK9 Inhibitor.

## Coronary artery calcification: entry to new frontier of PCSK9 inhibitors

Today, rapid aging in society is making progress in developed countries. One of the most serious health problems in an aging society is atherosclerosis, of which final phase is coronary artery calcification (CAC). CAC could increase the risk of complications during coronary catheterization and coronary artery bypass surgery as well as ischemic heart disease (IHD). We, members in the aging society, would see much more patients with CAC in the near future. We, however, have no medications for CAC today. Moreover, statins that are used in the world have been known for CAC formation after long-term use. The team led by Yuichi Ikegami and Ikuo Inoue in Japan’s Saitama Medical University found that CAC in patients with atherosclerosis is improved by PCSK9 inhibitors compared to statins by using coronary computed tomographic angiography. This study shows us a hint that could lead us to new preventive therapies of IHD.

## Introduction

Recent studies reported that protein convertase subtilisin/kexin type 9 (PCSK9) inhibitor therapy^1^ reduced adverse events by decreasing low-density lipoprotein (LDL) levels. Various image analysis methods are employed to identify patients at risk of coronary artery disease (CAD) events before their onset. Serial intravascular ultrasonography (IVUS), an invasive method for the detection of CAD events, revealed that PCSK9 inhibitor exerted favorable effects against the progression of coronary atherosclerosis.^2^ Moreover, coronary computed tomographic angiography (CCTA) has recently emerged as an accurate non-invasive method for the detection of coronary atherosclerosis and exclusion of obstructive CAD.^3^ Unlike IVUS, CCTA is a non-invasive method that is easy to use in outpatient clinics. It also permits the visualization of the coronary artery calcium (CAC) score beyond direct luminal diameter stenosis including graded measures of coronary plaque composition.

CAC score is a strong marker of coronary events^4^ and is a risk factor for atherosclerotic complications.^5^ The statin therapy^6^ reduces CAD events by decreasing LDL levels. However, the findings of recent studies with large sample sizes suggested that statins promote coronary vascular calcification.^7,8^ In contrast, other studies demonstrated that statins protect against coronary vascular calcification.^9–11^ The extent of LDL-cholesterol (LDL-C)-lowering effects may modulate coronary vascular calcification.

As more powerful and novel medications, PCSK9 inhibitors have safely been administered to patients worldwide. Alirocumab (Praluent, Regeneron/Sanofi), a type of PCSK9 inhibitor and a human immunoglobulin G1 monoclonal antibody (mAb), mediates the proteolytic degradation of hepatic LDL receptors (LDLR), resulting in the more efficient clearance of apolipoprotein B (ApoB)-containing lipoproteins.^12^ Evolocumab (Repatha, Amgen), the other type of PCSK9 inhibitor and a human immunoglobulin G2 mAb, inhibits PCSK9 using the same mechanism as that of alirocumab.^13,14^ Moreover, the addition of evolocumab to the statin therapy achieved serum LDL-C levels of less than 30 mg/dL.^1,15^ The effects of PCSK9 inhibitors, which have potent LDL-lowering activities, on vascular calcification have been of attracting interest. However, it currently remains unclear whether PCSK9 inhibitors modulate coronary atheroma calcification in vivo in humans.

The aim of the present study was to retrospectively evaluate the relationship between statin and/or PCSK9 inhibitor therapy and CAC score in patients undergoing CCTA.

## Results

Univariate and multivariate analyses for CAC score in the unpaired cross-sectional study (*n* = 120).

There were no adverse events during this clinical study. Forty-seven (47%) patients had a CAC score >0, 15 (12.5%) had severe coronary artery calcification (CAC score >400 AUs), while no patient had extensive coronary calcification with a CAC score >1000 AUs. The relationships between CAC score and each clinical variable were initially examined using Univariate and multivariate analyses (Table 1). A correlation was only observed between CAC score and lipid-lowering drug use (*P* < 0.0001). No correlations were found between CAC score and several traditional risk factors for CAD such as hemoglobin A1c (A1c) levels, blood pressure, and lipid levels. The occurrence of CAC score significantly increased with age (*P* = 0.048), particularly when patients aged >60 years were compared with those aged between 35 and 50 years (data not shown). CAC score was also slightly more common in males than in females (*P* = 0.20), in patients using antihypertensive drugs than in those without (*P* = 0.07), and in patients using insulin than in those without (*P* = 0.0001).

**Table 1:** The *p* value of univariate and multivariate analyses for associations between coronary artery calcium score and each data (*n* = 120)

	Univariable	Multivariable
Age	0.048	0.1010
Sex	0.76	
Body height	0.83	
Body weight	0.38	
Body mass index	0.32	0.6052
Aspartate aminotransferase	0.08	
Alanine aminotransferase	0.053	
Blood urea nitrogen	0.12	
Creatinine	0.64	
Creatine phosphokinase	0.77	
Total cholesterol	0.79	
Triglyceride	0.31	0.5375
HDL-C	0.16	0.2658
LDL-C	0.26	0.4951
Lp(a)	0.59	0.1561
Hemoglobin A1c	0.5	0.1541
Fasting blood glucose	0.17	0.1053
Systolic blood pressure	0.31	
Diastolic blood pressure	0.41	
Antihypertensive drug use	0.06	0.0120
Lipid lowering drug use	<0.0001	0.0050

The coronary artery calcium score was the log-transformed data

### Patient backgrounds in three groups in the unpaired cross-sectional study

In the unpaired cross-sectional study, we divided 120 patients into three groups: no-statin-no-PCSK9 group, *n* = 41; statin group, *n* = 60; statin; and PCSK9 group, *n* = 19. Table 2 shows the clinical characteristics of the patient backgrounds of the three groups. Recruitment was balanced for age, Body Mass Index (BMI), rate of hypertension treatment, blood pressure, plasma glucose, and A1c among patients in the three groups.

**Table 2:** Baseline characteristics of the unpaired cross-sectional study

	No-statin-No-PCSK9 group	Statin group	PCSK9 group	*P*-value
	(*n* = 41)	(*n* = 60)	(*n* = 19)	
Mean age (year)	64.6 ± 10.7	65.7 ± 5.0	64.1 ± 7.9	0.7738
Male sex (%)	51	65	32	0.0310
BMI (kg/m^2^)	24.0 ± 4.3	24.8 ± 3.6	24.2 ± 2.7	0.6065
Rate of hypertension treatment (%)	27	30	26	0.9298
Smoking (%)	7	3	0	0.0456
Past history of coronary heart disease (%)	13	39	56	0.0010
Family history of coronary heart disease (%)	20	37	47	0.0572
Systolic bood pressure (mmHg)	135 (91–170)	134 (90–168)	141 (94–164)	0.9201
Diastolic bood pressure (mmHg)	84 (60–100)	83 (59–99)	86 (58–98)	0.9104
Plasma glucose (mg/dL)	124.3 ± 41.0	127.8 ± 41.7	121.0 ± 28.6	0.8200
Hemoglobin A1c (%)	6.14 ± 0.94	6.28 ± 0.81	6.09 ± 0.80	0.7218
Total cholesterol (mg/dL)	201.2 ± 57.5	178.4 ± 46.5	176.7 ± 46.4	0.1829
Triglyceride (mg/dL)	163.8 ± 99.5	192.7 ± 237.4	188.1 ± 96.0	0.7974
High density lipoprotein-cholesterol (mg/dL)	54.7 ± 13.3	54.7 ± 12.4	58.7 ± 12.8	0.5125
Low density lipoprotein-cholesterol (mg/dL)	114.8 ± 53.7	91.4 ± 37.9	79.2 ± 41.6	0.0213
Lp(a) (mg/dL)	7.8 ± 9.7	22.4 ± 22.6	26.0 ± 33.1	0.4678

Data were average ± standard deviation or median (range of 10th–90th percentile)

### CAC score of the three groups in the unpaired cross-sectional study

**Fig. 1:**
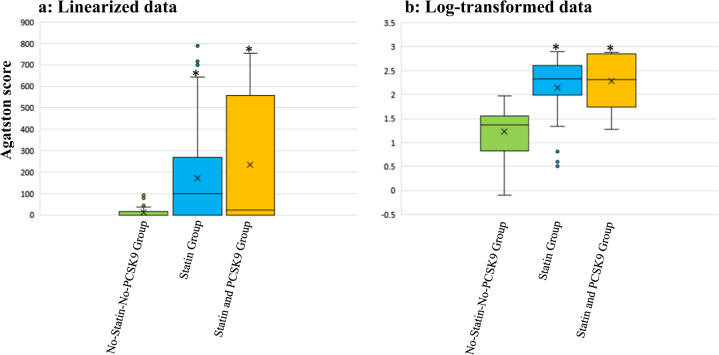
**a**, **b** Coronary artery calcium score (Agatston score) of the three groups in the unpaired cross-sectional study (*n* = 120). Data were expressed as the median and 10th–90th percentile. The horizontal line inside the box is the median. Boxes represent the interquartile range (25th–75th percentile). Whiskers represent the 10th–90th percentile. The x marks in the box indicate the average. **a** Linearized data, **b** Log-transformed data. *P < 0.05 vs. no-statin-no-PCSK9 group by a one-way analysis of variance

CAC scores were also log-transformed to adjust for a skewed distribution. Figure 1 shows the linearized data (Fig. 1a) and log-transformed data (Fig. 1b) for CAC score in the three groups. CAC score was significantly higher in the statin group, and in the statin and PCSK9 group than in the no-statin-no-PCSK9 group. CAC score was high in the statin group, and the statin and PCSK9 group because statin and/or PCSK9 inhibitor users had a higher CAD burden.

### Changes in the CAC score in the paired longitudinal study

**Fig. 2:**
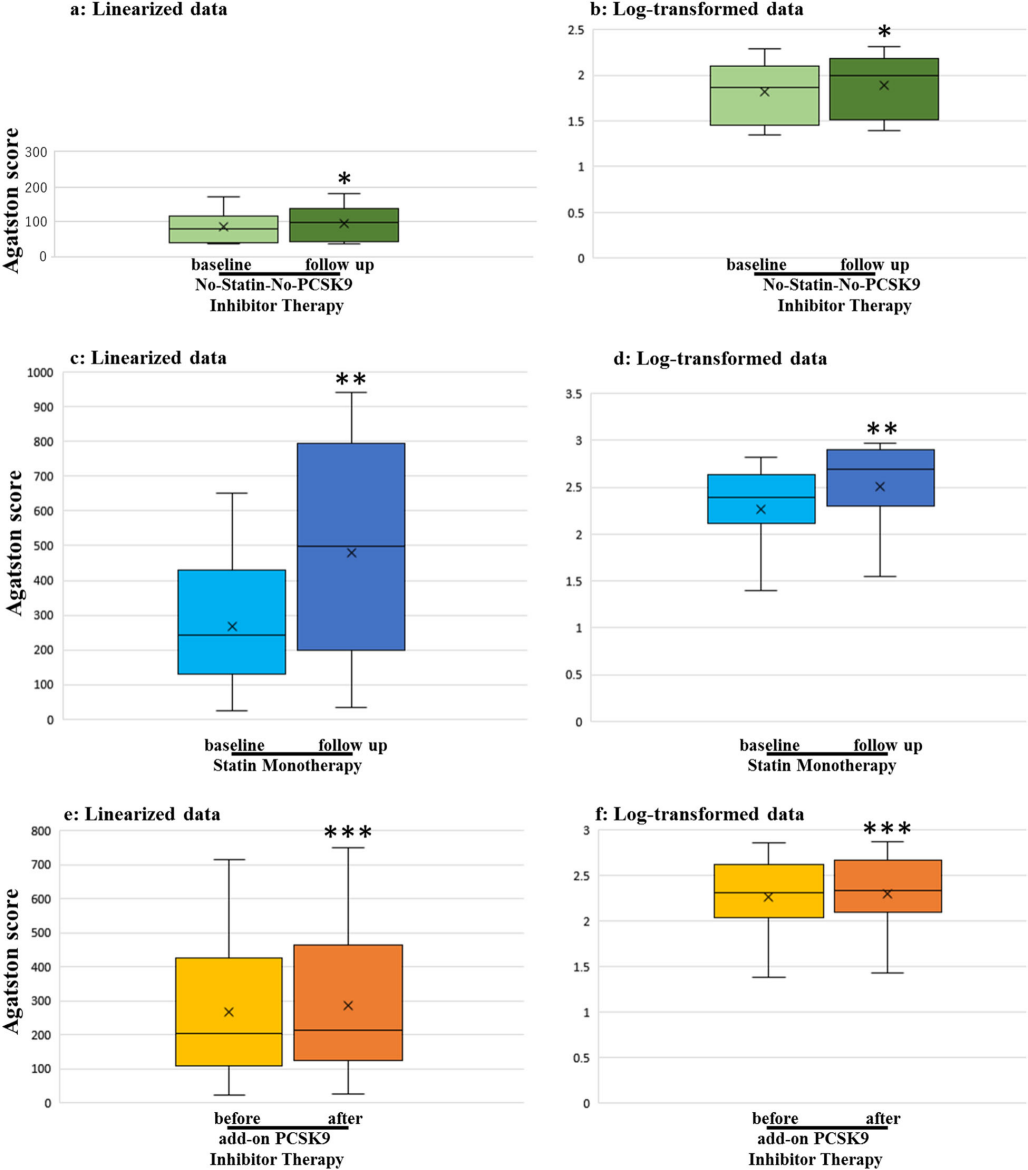
Changes in coronary artery calcium score (Agatston score) at the baseline and at follow-up for the duration of no-statin-no-PCSK9 inhibitor therapy (**a**, **b**) (*n* = 10) and in monotherapy (**c**, **d**) (*n* = 15) in the paired longitudinal study. Changes in coronary artery calcium Score (Agatston score) before and after add-on PCSK9 inhibitor to the statin therapy (**e**, **f**) (*n* = 16) in the paired longitudinal study. Data were expressed as the median and 10th–90th percentiles. The horizontal line inside the box is the median. Boxes represent the interquartile range (25th–75th percentile). Whiskers represent the 10th–90th percentile. The x marks in the box indicate the average. **a**, **c**, **e**: linearized data, **b**, **d**, **f**: log-transformed data. *P < 0.05 vs. baseline for the no-statin-no-PCSK9 inhibitor therapy by the non-parametric Wilcoxon signed-rank test. **P < 0.01 vs. baseline for the statin monotherapy by the non-parametric Wilcoxon signed-rank test. ***P < 0.05 vs. before add-on PCSK9 inhibitor to statin therapy by the non-parametric Wilcoxon signed-rank test

Table 3 shows the clinical characteristics of patients backgrounds of the three groups: no-statin-no-PCSK9 group, *n* = 10; statin monotherapy group, *n* = 15; add-on PCSK9 inhibitors therapy group, *n* = 16. Recruitment was balanced for age, BMI, and rate of past history of CHD, family history of CHD, and smoking among patients in the three groups. The rate of heterozygous familial hypercholesterolemia (HeFH) was significantly lower in the no-statin-no-PCSK9 group. There was no homozygous or compound heterozygous familial hypercholesterolemia in the three groups. CAC progression was also calculated as [log(CAC) at the follow-up]–[log(CAC) at the baseline]. Figure 2 shows changes in the linearized data (Fig. 2a) and log-transformed data (Fig. 2b) for CAC score at the baseline and follow-up for the no-statin-no-PCSK9 group. The no-statin-no-PCSK9 group contains many statin-intolerant patients. Figure 2 shows changes in the linearized data (Fig. 2c) and log-transformed data (Fig. 2d) for CAC score at the baseline and follow-up for the duration of statin monotherapy. The mean dose duration of statin monotherapy was 2.79 years. Figure 2 shows the changes in the linearized data (Fig. 2e) and log-transformed data (Fig. 2f) for CAC score before and after add-on PCSK9 inhibitors therapy. The mean dose duration of statin add-on PCSK9 inhibitor therapy was 0.75 years. Values are shown as medians (range of the 10th–90th percentile). The changes in CAC score significantly increased with the administration of statins and/or PCSK9 inhibitors (*P* < 0.05 and *P* < 0.01, respectively).

**Table 3:** Baseline characteristics of the paired longitudinal study

	No-Statin-No-PCSK9 inhibitor therapy (*n* = 10)	Statin monotherapy (*n* = 15)	Add-on PCSK9 inhibitor therapy (*n* = 16)	*P* value
Mean age (yr)	65.3 ± 8.6	65.3 ± 8.6	66.1 ± 9.1	0.9887
Male sex (%)	50	53	50	0.9846
BMI (kg/m^2^)	24.8 ± 2.5	23.8 ± 2.6	24.7 ± 3.9	0.9847
Smoking (%)	0	7	0	0.9400
Past history of coronary heart disease (%)	60	67	63	0.9586
Family history of coronary heart disease (%)	30	27	38	0.8705
Heterozygous familial hypercholesterolemia (%)	20	80	81	0.0170

Data were average ± standard deviation. The *p*-value was determined by using Kruskal–Wallis test

### Annual CAC score progression in the paired longitudinal study

**Fig. 3:**
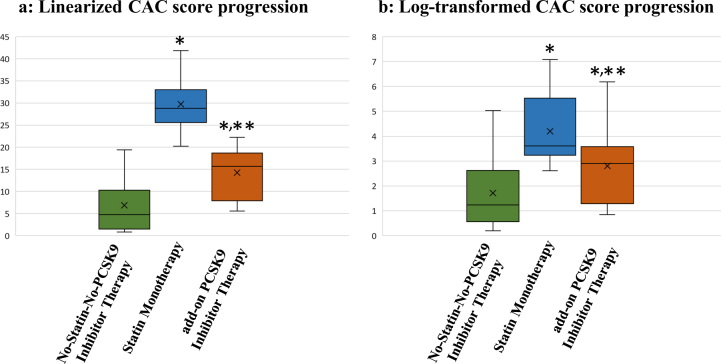
**a**,**b**: Comparison of annual percentage change in coronary artery calcium score (CAC) between no-statin-no-PCSK9 inhibitor therapy (*n* = 10), statin monotherapy (*n* = 15), and add-on PCSK9 inhibitor therapy in the paired longitudinal study (*n* = 16). Data were expressed as the median and 10th–90th percentiles. The horizontal line inside the box is the median. Boxes represent the interquartile range (25th–75th percentile). Whiskers represent the 10th–90th percentile. The x marks in the box indicate the average. **a** linearized CAC score progression, **b** log-transformed CAC score progression. *P < 0.05 vs. no-statin-no-PCSK9 inhibitor therapy and **P < 0.05 vs. statin monotherapy by the non-parametric Mann–Whitney *U*-test

CAC progression was also calculated as [log(CAC) at the follow up]-[log(CAC) at the baseline], and was then retransformed to depict the percentage change in CAC (Fig. 3). Annual linearized CAC score progression was 29.7% with statin monotherapy and 14.3% with PCSK9 inhibitor added to statin therapy (Fig. 3a). Annual log-transformed CAC score progression was 4.2% by statin monotherapy and 2.8% with PCSK9 inhibitor added to statin therapy (Fig. 3b). Annual CAC score progression was significantly smaller by PCSK9 inhibitor added to statin therapy than by statin monotherapy (*P* < 0.01) (Fig. 3).

Moreover, each clinical value was also calculated as [value at the follow-up]–[value at the baseline] and was then re-transformed to depict the percentage change in the value. A multivariate analysis was performed to identify the risk factors of annual CAC score progression rate. Potential confounding factors included in the model were: Triglyceride, HDL-C, LDL-C, Lp(a), hemoglobin A1c, fasting blood glucose, systolic blood pressure, and diastolic blood pressure retrieved. In the case of PCSK9 inhibitor added to the statin therapy, the *P* value of the multivariate analyses for associations between the log-transformed annual CAC score progression rate and each data were: Triglyceride 0.7454, HDL-C 0.9476, LDL-C 0.8751, Lp(a) 0.5421, hemoglobin A1c 0.5831, fasting blood glucose 0.3679, systolic blood pressure 0.2665, and diastolic blood pressure 0.5685. In the case of statin monotherapy therapy, the *P* value of multivariate analyses for associations between the log-transformed annual CAC score progression rate and each data were: Triglyceride 0.5781, HDL-C 0.6427, LDL-C 0.8991, Lp(a) 0.8546, hemoglobin A1c 0.4456, fasting blood glucose 0.2240, systolic blood pressure 0.4687, and diastolic blood pressure 0.6697. Thus, the results of multivariate analysis indicated that there was a statistically significant difference in annual CAC score progression rate.

## Discussion

We identified a strong relationship between statin and/or PCSK9 inhibitor therapy and CAC score. Annual CAC score progression was 29.7% by statin monotherapy and 14.3% by PCSK9 inhibitor added to statin therapy. Our results indicated that the annual increase induced in CAC scores by adding PCSK9 inhibitors to statin was smaller than that by statin monotherapy. Our data showed that CAC might be prevented with PCSK9 inhibitors.

Computed tomography (CT) for calcium scoring is a simple and convenient test for the identification of CAD and is widely used worldwide. Raggi et al. reported that an annual calcium score progression of >15% was associated with a worse prognosis for each standard calcium score category.^16^ An annual increase in CAC >15% or in CAC >100 units is predictive of future myocardial events. Previous studies reported that CAC was associated with atherosclerotic complications. CAC is a strong marker of coronary events.^4^ The prevalence of CAC is dependent on age and gender, occurring in more than 90% of men and 67% of women older than 70 years of age.^17,18^ Additionally, individuals with a higher BMI, higher blood pressure, abnormal lipids (higher LDL-C or triglyceride (TG), lower high-density lipoprotein cholesterol (HDL-C), or use of lipid-lowering medication, glucose disorders (impaired fasting glucose, untreated, or treated diabetes mellitus), a familial history of CAC and chronic kidney disease (CKD), higher fibrinogen level, and higher C-reactive protein (CRP) level were found to be more susceptible to CAC.^19^

Our results are inconsistent with previous findings. The present results indicated that the occurrence of CAC increased with hypolipidemic drug use only (*P* < 0.0001) (Table 1). This may be explained by the high CAC scores of patients treated for hyperlipidemia due to the small sample size. In addition, a hypolipidemic drug is likely to have been administered to arteriosclerosis patient with high CAC scores. We did not find a medical therapy targeting the reductions in CAC from univariable analyses (Table 1). Contrary to expectations, the results of the unpaired cross-sectional study suggested that statin and/or PCSK9 inhibitor therapy is an independent predictor of a high CAC score, indicating that statins and/or PCSK9 inhibitors may promote vascular calcification (Fig. 1).

Since our unpaired cross-sectional study was unable to confirm whether statin and/or PCSK9 inhibitor therapy increased the CAC scores, longitudinal studies were performed over a long period. Our results indicated that the statin (Fig. 2c, d) and/or PCSK9 inhibitor (Fig. 2e, f) therapy promoted coronary atheroma calcification. However, our longitudinal results indicated that the annual CAC score progression rate was 29.7% by statin monotherapy and 14.3% by PCSK9 inhibitor added to statin therapy (Fig. 3a). Our data indicated that CAC might be prevented with add-on PCSK9 inhibitors to statin.

The findings of recent studies with larger sample sizes also suggest that statins promote vascular calcification.^7,8^ Henein et al. reported an annual CAC score increase of 30% at the age of 50 and 21.5% at the age of 70 by statin therapy. These findings were consistent with our present results. Moreover, in vivo IVUS showed that statins promote plaque calcification, which may explain their stabilizing effects.^20^ Statins have been suggested to stabilize plaques by decreasing lipid-rich and necrotic plaque components, but increasing plaque calcification.^20,21^ Therapy with evolocumab, a PCSK9 inhibitor, was recently shown to reduce the percent atheroma volumes (−0.95%) with plaque regression^2^ using serial IVUS imaging, suggesting the ability of PCSK9 therapy to affect coronary artery plaque composition. Grayscale IVUS imaging enables qualitative assessments of atherosclerotic plaques.^22^ Further studies need to focus on how to change coronary plaque composition assessed by greyscale IVUS, with PCSK9 inhibitor therapy.

The following paradoxical results have been obtained:^23^ (1) CAC progression is an independent predictor of CAD events; (2) statins and/or PCSK9 inhibitors promote plaque regression; and (3) statins promote CAC progression (Fig. 3). Recently, it has been reported that patients with highest PCSK9 concentrations had the highest CAC score.^24^ Plasma PCSK9 concentrations were also independently associated with the severity of coronary artery stenosis and even major adverse cardiovascular events in stable CAD patients.^25,26^ Statins decrease hepatic intracellular cholesterol, resulting in increased PCSK9 protein, as well as increased LDLR.^27^ It is possible that PCSK9 inhibitors canceled the action of PCSK9 increased by statin treatment, resulting in the prevention of calcification.

It is important to consider estimating CAC progression. Liu W et al.^28^ recently reviewed whether the high prevalence of CAC in CHD patients makes percutaneous coronary intervention (PCI) difficult to perform. Coronary calcification increases the risk of procedural complications such as dissection, thrombosis, and restenosis.^29–31^ Blood vessels with advanced calcification are less likely to respond successfully to bypass surgery. When statins were administered to patients with an annual increase in CAC of >15% or annual increase of CAC >100 units, repeat CCTA was considered every 6 months. Large doses of radiation may cause serious tissue damage and increase the risk of developing cancer. Although CT for calcium scoring involves exposure to radiation, the effective dose is very small (~1.0 mSv).^23^ However, it is necessary to evaluate the accumulated radiation exposure dose by other CT, PET scans, or any other imaging modalities.

Although add-on PCSK9 inhibitor to statin is one of the options to prevent CAC, medical and pharmaceutical advances are critical. Specifically, a new class of drug therapy^32^ should be developed by elucidating the progression mechanisms of CAC for its recovery and repair for anti-aging. Further studies should investigate whether PCSK9 inhibitor is the anti-aging drug through this pleiotropic mechanism.

### Limitations

The present study had several limitations, because the differences in the dose and the products of statins have significant influence on the results, it will be necessary to evaluate the effect of each statin. The enrolled number of subjects was very small and this study had an open-label design. The markedly short study duration may also be a limitation. The treatment period may have been too short to obtain a complete therapeutic effect. Therefore, a long-term, larger-scale, and double-blinded study in which the drugs are administered to patients with high risk hyperlipidemia is needed in the future. Furthermore, a selection bias may be present. This study retrospectively evaluated the relationship between statin and/or PCSK9 inhibitor therapy and CAC score in patients undergoing CCTA. Thus, a prospective study is needed in future. Another limitation is that CCTA imaging cannot perform qualitative assessments of atherosclerotic plaques. Methods that provide information on the composition of plaques need to be considered. In addition, we excluded subjects with TG > 400 mg/dL because we calculated the LDL-C level using the Friedewald equation. Moreover, all participants were Japanese, therefore our results cannot be generalized to other races or ethnic groups.

## Conclusion

Our results indicated that the annual increase in CAC was smaller by add-on PCSK9 inhibitors than by statins. The progression of CAC scores by statin monotherapy was 29.7%, while that by combination therapy with a PCSK9 inhibitor and a statin was 14.3%. Our data showed that CAC might be prevented with PCSK9 inhibitors.

## Methods

### Subjects

This study was parallel, retrospective in nature, and was divided into two parts; an unpaired cross-sectional study and paired longitudinal study. In the unpaired cross-sectional study, we enrolled 120 patients receiving CCTA and divided them into three groups: (1) neither statin nor PCSK9 inhibitor therapy (no-statin-no-PCSK9 group, *n* = 41), (2) statin monotherapy (statin group, *n* = 60), and (3) combination therapy with a PCSK9 inhibitor and a statin (statin and PCSK9 group, *n* = 19). In the paired longitudinal study, CCTA was repeated and compared for the duration of statin therapy in 15 patients. CCTA was repeated and compared before and after the addition of a PCSK9 inhibitor to the ongoing statin therapy in 16 patients. We enrolled the completely different patients for an unpaired cross-sectional study and a paired longitudinal study. The participants were provided written informed consent to participate in the study. As the present study is retrospective, we didn’t obtain them from all participants.

All patients visited the outpatient clinic of Saitama Medical University Hospital Endocrinology/Diabetes Department between July 2016 and July 2017 for the unpaired cross-sectional study and between July 2012 and April 2018 for the paired longitudinal study. For the cross-sectional and longitudinal study, statin and/or PCSK9 inhibitor was administered to patients (1) with CAD, (2) with HeFH according to the “2017 Guidelines for the Treatment of Dyslipidemia” by the Japan Atherosclerosis Society (JAS). Alirocumab was additionally administered at a dose of 75 or 150 mg once every 2 or 4 weeks. Evolocumab was additionally administered at a dose of 140 mg once every 2 or 4 weeks. All participants were treated with a stable dose of statins for at least one year prior to the investigation by CCTA. The LDL-C levels of the patients who received PCSK9 inhibitor, were higher than the target level recommended by the guidelines.

### Laboratory test

In laboratory tests on serum lipids, blood was collected in early in the morning after fasting. Plasma total cholesterol levels (TC) and TG were measured by enzyme methods, while HDL-C was measured using a selective solubilization method at the facilities of Saitama Medical University. LDL-C levels were calculated by the method of Friedewald (F method), and patients with TG of 400 mg/dL or higher were excluded.

### CT scan

Most patients have clinical symptoms such as chest pain and the ischemic change in electrocardiogram, which are suspecting symptoms of coronary arteriosclerosis. Thus, CCTA, an accurate non-invasive approach, was carried out twice to diagnose whether fatty deposits or calcium deposits were present in the coronary arteries. CAC was assessed by CT performed on a 64-channel detector scanner (SOMATOM Definition Flash 128, Siemens, Germany; SCENARIA 128) in the cine and straight mode. Scans were assessed by the electrocardiogram (ECG)-gated method, and a standard non-contrast protocol was used with a tube voltage of 120 kV, tube current of 50–80 mA, rotation time of 280 ms, slice thickness of 3 mm, and display field of view (DFOV) of 20 × 20 cm. CAC data were processed and analyzed using SYNAPSE VINCENT (FUJIFILM). These CAC data were blinded and evaluated by a single radiologist. SMARTSCORE 4.0 (GE Healthcare) was used to assess the CAC scores. Plaque measurement software automatically recognized the plaques and lumens, and vessel walls and plaque edges were then manually modified where needed in order to define both ends of the plaques.

### CAC

CAC was quantified as a lesion with an area >1 mm^2^ and peak intensity >130 Hounsfield Units (HUs)^33^ based on the Agatston method previously described in detail and expressed in Agatston units (AUs).^34^ The total CAC score was calculated as the sum of the CAC scores in the left main artery (LM), the left anterior descending artery (LAD), the left circumflex artery (LCX), and the right coronary artery (RCA). Since a CAC score >100 AUs is associated with an increased risk of myocardial ischemia and coronary heart disease (CHD)-related events,^35^ we used this threshold to identify patients with a definite to extensive plaque burden. It has been reported that CAC score >400 AUs were related to heart failure.^33^

### Exclusion criteria

We used our previous criteria^15^ that we describe in detail. Major exclusion criteria were as follows: (1) patients treated with glucagon-like peptide 1 (GLP-1) receptor agonists, (2) CKD (eGFR < 45 mL/min), (3) fasting TG greater than or equal to 400 mg/dL, (4) blood pressure >180/110 mmHg, (5) A1c that changed by more than 2% within 3 months, (6) severe ketosis, diabetic coma or precoma, peripheral artery disease, abdominal aortic aneurysm, carotid artery occlusion >50% without symptoms, carotid endarterectomy, carotid artery stent procedure, renal artery stenosis, or renal artery stent procedure, (7) severe infection before and after surgery, patients determined to be ineligible for the trial by the investigator or a doctor and those suffering from serious trauma, (8) pregnant or breast-feeding women, (9) women of childbearing potential with no effective contraceptive method, (10) participants not previously instructed on a cholesterol-lowering diet in the first visit who were being treated with a stable dose of statins for at least 6 weeks prior to screening.

### Ethics statement

This study was conducted in accordance with the Good Clinical Practice (GCP), International Conference on Harmonization Guidelines (ICH), and applicable laws and regulations. The study protocol was approved by the Ethics Committee of Saitama Medical Hospita (Approved No. 17–041). Our study was a retrospective study, which was simply look back in time and collect the data. It was posted on the official website of Saitama Medical University Hospital as a widely well-known method of research contents. If there was an offer that the patient did not want to participate in the study, we removed the relevant information. We collected the information that has been recorded from April 1, 2011 to January 13, 2018.

### Statistical analysis

Measurement values were expressed as means ± SD or medians (range of the 10th–90th percentile). Statistical analyses were performed using JMP® ver.7.0 (SAS Institute, Inc., Cary, NC, USA). Univariate and multivariate analyses were performed on clinical variables to CAC scores. CAC scores were compared by a one-way analysis of variance with significance levels of 5% in three groups. CAC scores of the baseline and follow-up of statin and/or PCSK9 inhibitor therapy were compared with the non-parametric Wilcoxon signed-rank test with significance levels of 5%. Annual CAC score progression rate by statin monotherapy and by PCSK9 inhibitor added to statin therapy was compared with the non-parametric Mann–Whitney *U*-test with significance levels of 5%. In addition, the multivariate analysis was conducted to prove the significant different of the annual CAC score progression rate.

### Data availability

The data are available with an appropriate material transfer agreement. All relevant data are available from the authors. The trial number is UMIN000006539, and the registry URL: https://upload.umin.ac.jp/cgi-open-bin/ctr/ctr_view.cgi?recptno=R000007750. And the trial number is UMIN000018818, and the registry URL: https://upload.umin.ac.jp/cgi-open-bin/ctr/ctr_view.cgi?recptno=R000021766.
